# Dysfunctional information processing during an auditory event-related potential task in individuals with Internet gaming disorder

**DOI:** 10.1038/tp.2015.215

**Published:** 2016-01-26

**Authors:** M Park, J-S Choi, S M Park, J-Y Lee, H Y Jung, B K Sohn, S N Kim, D J Kim, J S Kwon

**Affiliations:** 1 Department of Psychiatry, SMG-SNU Boramae Medical Center, Seoul, Republic of Korea; 2 Department of Psychiatry and Behavioral Science, Seoul National University College of Medicine, Seoul, Republic of Korea; 3 Department of Psychiatry, Seoul St. Mary's Hospital, The Catholic University of Korea College of Medicine, Seoul, Republic of Korea

## Abstract

Internet gaming disorder (IGD) leading to serious impairments in cognitive, psychological and social functions has gradually been increasing. However, very few studies conducted to date have addressed issues related to the event-related potential (ERP) patterns in IGD. Identifying the neurobiological characteristics of IGD is important to elucidate the pathophysiology of this condition. P300 is a useful ERP component for investigating electrophysiological features of the brain. The aims of the present study were to investigate differences between patients with IGD and healthy controls (HCs), with regard to the P300 component of the ERP during an auditory oddball task, and to examine the relationship of this component to the severity of IGD symptoms in identifying the relevant neurophysiological features of IGD. Twenty-six patients diagnosed with IGD and 23 age-, sex-, education- and intelligence quotient-matched HCs participated in this study. During an auditory oddball task, participants had to respond to the rare, deviant tones presented in a sequence of frequent, standard tones. The IGD group exhibited a significant reduction in response to deviant tones compared with the HC group in the P300 amplitudes at the midline centro-parietal electrode regions. We also found a negative correlation between the severity of IGD and P300 amplitudes. The reduced amplitude of the P300 component in an auditory oddball task may reflect dysfunction in auditory information processing and cognitive capabilities in IGD. These findings suggest that reduced P300 amplitudes may be candidate neurobiological marker for IGD.

## Introduction

The increasing popularity of the Internet has led to a growing body of research in various fields related to Internet addiction, gaming addiction and pathological Internet use.^1, 2^ Excessive Internet use or Internet gaming may become out of control and lead to serious impairment in cognitive, psychological and social functioning, and these potential risks of Internet use have been increasingly recognized as significant mental health issues in the international community.^3^ In 2013, the American Psychiatric Association (APA) included Internet gaming disorder (IGD) in section 3 (Emerging measures and models) of the *Diagnostic and Statistical Manual of Mental Disorders*, fifth edition (DSM-5) as a condition for further study.^4^ However, the American Psychiatric Association noted a lack of standard diagnostic criteria and the need for further research. Additional studies to define the features of IGD, to obtain cross-cultural data on the reliability and validity of specific diagnostic criteria and to elucidate its associated biological features are needed before IGD is included in the next version of the DSM as a formal disorder.^5^

People playing Internet games for a long period of time are repeatedly exposed to visual and auditory events, and this continuous exposure to colorful images and dynamic sounds may cause visual or auditory fatigue and problems in related brain regions.^6, 7^ Moreover, recent neuroimaging studies have reported significant changes in brain function and structure associated with IGD.^8, 9, 10^ According to previous research, patients with IGD have reduced regional homogeneity in the superior temporal gyrus at rest.^11, 12^ The superior temporal gyrus, which contains the primary auditory cortex, is thought be important for integrating auditory and visual information.^13, 14, 15^

Event-related potential (ERP) measures have been used extensively to investigate brain functions and the neural mechanisms of attention and cognition due to their sensitive temporal resolution and noninvasiveness.^16, 17, 18^ P300 component of ERP is a large positive deflection that occurs ~300–500 ms after stimuli onset and has maximal amplitude over in the central and parietal regions of the scalp. It is thought to reflect selective attention, memory or processing of incoming information, and has been reported to be reduced in amplitudes in substance use disorder by the majority of studies.^19^ Several previous studies have provided evidence regarding substance use disorder in the form of ERP data collected during the performance of auditory oddball tasks. Alcoholics showed a significant reduction in the amplitudes of the P300 component, which is deficient in alcoholism, while performing an auditory oddball task.^20, 21, 22^ Some findings have showed that reduced P300 amplitudes have been observed in individuals at risk for alcoholism, which suggested a lack of capacity to allocate neural resources for encoding specific events and could be due to impaired cortical functions.^23, 24^ Studies on smoking dependence have also demonstrated a reduction in P300 amplitudes during an auditory oddball task in smokers compared with controls,^25, 26^ and Moeller *et al.*^27^ reported that lower P300 amplitudes were found in cocaine users than in controls.

To our knowledge, no prior study has tested the pattern of the P300 component in patients with IGD using an auditory oddball task, and only a few studies have used ERP methods to examine the features of IGD.^28, 29^ For example, Dong *et al.*^30^ used a go/no-go task to study response inhibition in people with Internet addiction disorder. In particular, as mentioned above, patients with IGD are repeatedly exposed to various kinds of visual and auditory stimulations such that it is necessary to investigate neural functions associated with information processing in IGD. The present study compared the ERP patterns associated with auditory information processing in patients with IGD with those in healthy controls (HCs) to identify the neurophysiological features that may serve as possible biomarkers of IGD. We hypothesized that the P300 amplitudes of patients with IGD in response to target stimuli would be reduced compared with those of HCs. In addition, we hypothesized that there would be the relationship between P300 amplitudes and the severity of IGD symptoms.

## Materials and Methods

### Participants

Twenty-six patients with IGD and 23 age-, sex-, education- and intelligence quotient (IQ)-matched HCs participated in this study. All patients were seeking treatment at the outpatient clinics of SMG-SNU Boramae Medical Center in Seoul, South Korea, due to excessive participation in Internet gaming. The Institutional Review Board of the SMG-SNU Boramae Medical Center approved the study protocol, and all subjects provided written informed consent before participation. A clinical interview by an experienced psychiatrist was administered for the diagnosis of IGD according to DSM-5 criteria, and Young's Internet Addiction Test (IAT)^31^ was used to assess the severity of the participants' disorder. In this study, a modified IAT made for assessing Internet games was used.^32^ To clarify the pathological changes associated with IGD, we included only those subjects with IAT scores of at least 70 (ref. 33) who spent more than 4 h daily and 30 h per week using Internet games, which restricted our sample to those with severe IGD, and excluded those who were merely at a high risk of developing this disorder due to excessive Internet gaming. In addition, the Structured Clinical Interview for DSM-IV was used to identify past and current psychiatric illnesses. Of 26 patients with IGD, 4 and 3 fulfilled DSM-IV criteria for depressive disorder and anxiety disorder, respectively. HCs were recruited from the local community and had no history of any psychiatric disorder. HCs played Internet games <2 h per day. The Beck Depression Inventory (BDI)^34^, the Beck Anxiety Inventory (BAI)^35^ and the Barratt Impulsiveness Scale-11 (ref. 36) were used to gather clinical data related to IGD.

Exclusion criteria were a history of significant head injury, seizure disorder, mental retardation, psychotic disorder and substance use disorder except nicotine. All participants were medication-naive at the time of assessments. The Korean version of the Wechsler Adult Intelligence Scale-III was administered to all subjects to estimate IQ, and we included only subjects with Wechsler Adult Intelligence Scale-III scores of at least 80.

### Task and procedure

We used the auditory oddball task, which involves presenting standard stimuli (85%) as well as rare, deviant stimuli (15%) in a pseudorandomized order at an 85-dB sound pressure level. Three hundred stimuli were presented binaurally by a STIM 2 sound generator (Compumedics, El Paso, TX, USA). Stimuli were presented under two different pitch conditions: the infrequent deviant stimulus was classified as a high-frequency tone (2000 Hz); and the frequent standard stimulus was classified as a low-frequency tone (1000 Hz). The duration of each tone was 100 ms (10-ms rise and fall times) with fixed intertrial intervals of 1250 ms. The participants were instructed to press a response-box button with their right hand as quickly and accurately as possible in reaction to only to high tones. All participants were given the opportunity to practice before the actual task started. Participants completed three blocks of 100 trials while seated in a comfortable chair.

### ERP recording

Electroencephalogram and electrooculogram data were recorded using a 64-channel Quick-cap system (Compumedics) that referred to the linked mastoid in an isolated sound-shielded room. The location of the ground channel was between FPz and Fz. Horizontal and vertical electrooculograms were measured by electrodes placed at the outer canthus of each eye, and above and underneath the left eye, respectively. The electrical activities were continuously recorded at a sampling rate of 250, 500 or 1000 Hz. A band-pass filter was set to 0.3–100 Hz. Impedance at all recording electrodes was <10 kΩ.

### ERP analysis

Electrophysiological signals were further processed off-line using Curry 7 software (Compumedics). Recordings were first downsampled into 250 Hz. Data were then re-referenced against the common average reference and filtered with a frequency band pass from 0.3 to 30 Hz. Electroencephalogram and electrooculogram recordings were visually inspected to reject gross artifacts such as those involving movement. Eye blinks and eye movements were corrected based on the method of artifact reduction developed by Semlitsch *et al.*^37^ Data were then segmented into epochs of 1000 ms, which included the 100-ms pre-stimulus baseline period. Epochs with voltage in excess of ±70 μV were discarded automatically. Only trials with correct responses to deviant tones at four midline sites (FCz, Cz, CPz and Pz) were averaged and analyzed. Midline electrodes are commonly selected in oddball tasks investigating P300 components. The ERP waveforms for each participant had a minimum of 35 artifact-free trials. The P300 component was defined as the largest positive-going peak within the time window between 248 and 500 ms after stimulus onset. Topographic maps of P300 amplitudes were created using Scan 4.5 software (Compumedics).

### Statistical analysis

Demographic, clinical and behavioral data were analyzed with one-way analyses of variance (ANOVAs) or *χ*^2^-test, with the treating group (IGD and HC) as the between-subject factor. In terms of stimulus-locked ERP values, the amplitudes and latencies of the P300 component were separately analyzed with repeated-measures ANOVAs with electrode sites (FCz, Cz, CPz and Pz) as within-subject factors and group as the between-subject factor. In the case of sphericity violations, the lower-bound corrections were applied, and the corrected *P*-values were reported. P300 values associated with significant intergroup differences were subjected to analyses with clinical variables using two-tailed Pearson's correlation coefficients. Results with *P*-values <0.05 were regarded as significant. The variables showing significant main effects were further analyzed with *post hoc* comparisons using one-way ANOVAs. All statistical analyses were performed using SPSS v18.0 software (SPSS, Chicago, IL, USA).

## Results

### Demographic and clinical data

No significant group differences existed with regard to age, sex, education and estimated IQ. Patients with IGD had significantly higher scores on the IAT (F_(1, 47)_=450.99, *P*<0.001), BDI (F_(1, 46)_=49.92, *P*<0.001), BAI (F_(1, 46)_=11.17, *P*<0.01) and Barratt Impulsiveness Scale-11 (*F*_(1, 46)_=57.50, *P*<0.001) compared with HCs. The demographic and clinical characteristics of participants are presented in Table 1.

### Behavioral results

The accuracy rates of the two groups did not differ significantly. Although patients with IGD responded somewhat more slowly compared with the HCs, no significant group effects were observed. Behavioral performance data are presented in Table 2.

### ERP peak measures

**Figure 1:**
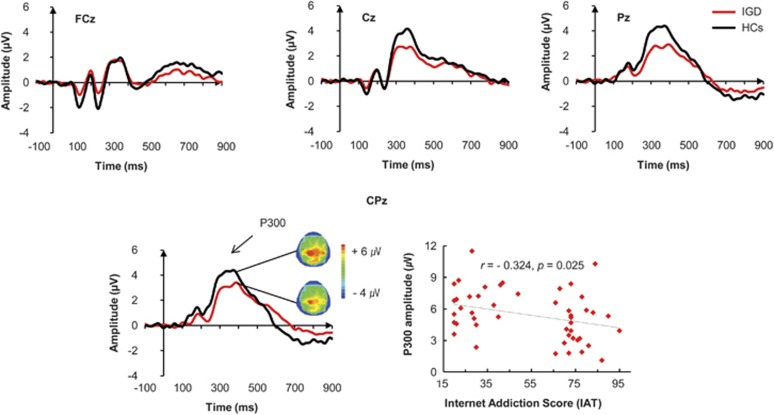
(Upper row) Grand-average event-related potential (ERP) waveforms over three electrode regions (FCz, Cz and Pz) in response to deviant tones in the auditory oddball task for patients with Internet gaming disorder (IGD) and healthy controls (HCs). (Lower row) Left-side figure indicates grand-average ERP waveforms at the midline centro-parietal electrode (CPz). Topographic maps indicate scalp distribution of P300 amplitude in two groups. Right-side figure represents correlations between Young's Internet Addiction Test (IAT) score and P300 amplitude at the midline centro-parietal electrode.

The grand-average ERP waveforms for deviant stimuli at four electrode sites are shown in Figure 1. Significant main effects of electrode site (F_(1, 45)_=16.73, *P*<0.001) and group (F_(1, 45)_=4.69, *P*=0.029) for P300 amplitudes were found. The P300 amplitude measured at CPz was the highest among the four electrode sites. No significant interaction was observed between the electrode site and group for the P300 amplitudes. Patients with IGD showed significantly lower P300 amplitudes than HCs at CPz (F_(1, 47)_=8.02, *P*<0.01) but not at FCz, Cz and Pz. In terms of P300 latencies, none of the main effects or interactions was statistically significant.

### Correlations between P300 amplitudes and clinical variables

Significant correlations were found between P300 amplitudes and IAT scores (Figure 1). IAT scores were significantly negatively correlated with P300 amplitudes at CPz (*r*=−0.324, *P*=0.025). No significant correlations were found between P300 amplitudes and BDI, BAI and Barratt Impulsiveness Scale-11 scores.

## Discussion

We investigated the electrical brain activity using an auditory oddball task in response to deviant stimuli. The auditory oddball task in this study may have been too easy, and behavioral performance did not differ significantly between patients with IGD and HCs. However, the present study found ERP differences between the two groups in an auditory oddball task. Therefore, ERP differences between groups were not due to differences in the behavioral performance but neurophysiological changes in the IGD group. Consistent with our prediction, the amplitude of the P300 component in response to deviant tones was diminished in patients with IGD compared with HCs at the midline centro-parietal electrode region. These reductions in the P300 amplitude in the auditory oddball task indicate that patients with IGD suffer from a dysfunction in auditory information processing and cognitive functioning. These results are consistent with previous ERP studies with individuals suffering from other addictions, who exhibited a decrement in the P300 amplitude.^19, 22, 38, 39^

P300 is considered to reflect the information-processing cascade associated with attentional and memory mechanisms. If the incoming stimulus in the oddball task is not the same and the subject allocates attentional resources to the target, the neural representation of the stimulus environment is updated and a P300 is elicited in addition to sensory potentials.^18, 40^ Therefore, the P300 component indexes fundamental attention- and memory-related operations. The correlational analysis revealed significant relationships between reduced P300 amplitudes and severe IGD symptoms. These results suggest that the P300 amplitude changes observed could be related to the clinical status of IGD and may be a candidate neurophysiological marker of IGD.

The neural generators of the P300 component have been widely investigated.^41, 42^ Although the precise neural origins of P300 are not clearly understood, some studies have consistently found that the P300 component is produced by a neural circuit pathway between the frontal and temporo-parietal regions.^43, 44^ The classic P300 component typically refers to the P3b elicited by target stimuli, whereas another subcomponent of the P300 is the P3a elicited by novel or nontarget stimuli. The P300 used in the present study refers to the P3b. The P300 component (or P3b) may originate from temporo-parietal regions, and the P3a may originate from frontal regions.^45, 46^ Kim *et al.*^12^ reported resting-state functional brain changes in the superior temporal gyrus and the posterior cingulate cortex in patients with IGD. As the posterior cingulate cortex is part of the default mode network as well as the parietal region, which is synchronized with low-frequency oscillations in some brain regions during the resting state, it is important for attention and self-monitoring as it involves cognitive functions associated with the coupling of executive control and the ability to disengage from the default mode network.^47^ The superior temporal gyrus is thought to be important in the processing of audiovisual information and is also among the key regions involved in the integration of auditory and visual cues, and in emotional perception based on auditory/visual information.^15^ The reduced P300 amplitudes in patients with IGD found in the present study may represent neurophysiological changes in the temporo-parietal regions, which are consistent with previous findings.^12^ In addition, this finding indicates that changes in P300 amplitudes may be associated with repeated exposure to various kinds of visual and auditory stimulations during playing Internet games, in patients with IGD.

Although the exact neurotransmitter systems underlying P300 generation remain unclear, some lines of evidence imply a neurotransmitter mediation of P300 generation. With regard to the P3b component, norepinephrine activity, which originates in the locus coeruleus, may contribute to P300 (or P3b) generation in humans.^48, 49^ On the other hand, Polich and Criado^50^ reported that the P300 amplitudes of controls and patients with restless leg syndrome were comparable, but they were greatly diminished in patients with Parkinson's disease, who have low levels of dopamine in the brain. Pogarell *et al.*^51^ also found that striatal dopamine D2/D3 receptor status was positively correlated with the P300 amplitudes in response to target tones in patients with depression. That is, reduced P300 amplitude is associated with reduced dopaminergic activity. Several previous studies have suggested that Internet addiction or IGD is associated with abnormalities in the dopamine reward system. Kim *et al.*^52^ found reduced levels of dopamine D2 receptor availability in subdivisions of the striatum, including the bilateral dorsal caudate and right putamen, in individuals with IGD. Abnormalities in the P300 amplitudes of patients with IGD may be indicative of impaired dopaminergic systems in IGD, which are commonly observed in other addiction disorders.^53^

The present study has several limitations. First, the sample used in this study was small, limiting the generalizability of the findings. Thus, future studies with larger samples are needed to more confidently identify the features of IGD. Although the number of participants in the study was small, we controlled for demographic characteristics such as age, sex, education, IQ and medication status. None of the participants was receiving medications. Electrophysiological activity can be affected by medications.^54, 55^ Therefore, our results excluded the effect of medication on ERPs. Second, patients with IGD had significantly higher scores on the BDI and BAI compared with HCs. To control potential confounding effects, analyses of covariance with BDI and BAI scores as covariates were performed on P300 amplitudes, and significant differences in P300 amplitudes still maintained between two groups. In addition, when we did analyses in individuals with IGD after excluding those with depressive disorder or anxiety disorder, the results were still significant. Moreover, we found no significant correlation between the P300 amplitude and the BDI and BAI scores. Third, the IAT scale used for the assessment of severity of IGD was a self-report form, which could lack objective characteristics. Fourth, a cross-sectional design was used in this study, but a longitudinal study observing the same participants over time would be more beneficial for elucidating the development of this disorder. Finally, we did not have clinical control group such as substance use disorder. In the further study, it is necessary to compare those in IGD with other addictive disorders in order to clarify the neurophysiological features specific to IGD. Despite these limitations, the findings of this study contribute to our understanding of alterations in the P300 component and the association of this component with the neuropsychological deficits associated with IGD.

In conclusion, our results reflected a reduction in the P300 amplitudes of the IGD group compared with those of the HC group during an auditory oddball task. Furthermore, the reduced amplitude of the P300 component was negatively correlated with the severity of IGD, which may represent deficits in auditory information processing and cognitive functions, as well as the relationship between this component and pathological Internet game use. The findings of the present study suggest that the reduction in P300 amplitude associated with brain functional abnormalities may be a candidate neurobiological marker for IGD, which may provide further insight into the neurophysiological mechanisms underlying this disorder. In order to identify whether alterations of P300 amplitudes in patients with IGD could be considered as a candidate trait maker or state maker, additional longitudinal studies and analysis of P300 amplitude in subjects at high risk for IGD are expected. When the abnormalities of P300 could be present in populations at high risk for IGD, P300 component of ERP could be regarded as a trait maker for IGD. In addition, when the abnormalities of P300 could be normalized along with symptom improvements after longitudinal assessments in patients with IGD, P300 indexes could be regarded as a state marker for IGD. Then, it could be used for assessing prognosis of IGD, or for prevention and early therapeutic interventions in patients with IGD.

## Figures and Tables

**Table 1:** Demographic and clinical characteristics of patients with IGD and HCs

	*IGD (*n=*26)*	*HCs (*n=*23)*	*F or* χ*^2^*	*d.f.*	P-*value*
*Demographic data*					
Age (years)	23.04±4.15	25.04±4.29	2.76	47	0.103
Sex (*n*; male/female)	20/6	20/3	0.82	1	0.365
Education (years)	14.12±1.90	14.87±1.60	2.22	47	0.143
Estimated IQ	117.62±13.12	123.87±9.29	3.62	47	0.063

*Clinical data*					
Age at onset of Internet use (years)	11.65±2.74	11.95±3.14	0.13	46	0.725
IAT***	76.35±7.14	28.43±8.64	450.99	47	0.000
BDI***	19.50±9.27	4.55±3.84	49.92	46	0.000
BAI**	18.12±12.82	8.05±6.43	11.17	46	0.002
BIS-11***	71.65±8.28	54.41±7.31	57.50	46	0.000

Abbreviations: BAI, Beck Anxiety Inventory; BDI, Beck Depression Inventory; BIS-11, Barratt Impulsiveness Scale-11; d.f., degrees of freedom; HCs, healthy controls; IAT: Young's Internet Addiction Test; IGD, Internet gaming disorder; IQ, intelligence quotient.

**P<0.01.

***P<0.001.

**Table 2:** Behavioral results (accuracy rates and reaction times) and ERP values (amplitudes and latencies of P300) in patients with IGD and HCs

	*IGD (*n=*26)*	*HCs (*n=*23)*	*F*	*d.f.*	P-*value*
*Behavioral results*					
Accuracy rate (%)	97.95±3.44	98.16±2.82	0.06	47	0.813
Reaction time (ms)	370.21±42.38	351.05±42.39	2.49	47	0.121

*ERP values*					
FCz amplitude (μV)	3.01±2.27	3.99±2.55	1.96	46	0.168
Cz amplitude (μV)	4.14±2.29	5.52±2.60	3.88	47	0.055
CPz amplitude (μV)**	4.52±2.42	6.36±2.08	8.02	47	0.007
Pz amplitude (μV)	4.47±2.70	5.76±1.96	3.53	46	0.067
FCz latency (ms)	366.31±86.43	402.82±98.38	1.87	46	0.178
Cz latency (ms)	389.62±70.31	359.22±51.99	2.90	47	0.095
CPz latency (ms)	376.92±62.08	345.65±52.04	3.60	47	0.064
Pz latency (ms)	375.28±59.52	350.26±60.41	2.09	46	0.155

Abbreviations: d.f., degrees of freedom; ERP, event-related potential; HCs, healthy controls; IGD, Internet gaming disorder.

**P<0.01.
